# Magnification effect on fine motor skills of dental students

**DOI:** 10.1371/journal.pone.0259768

**Published:** 2021-11-08

**Authors:** Danielle Wajngarten, Júlia Margato Pazos, Vinícius Perassoli Menegazzo, Juliana Pimentel Duarte Novo, Patrícia Petromilli Nordi Sasso Garcia

**Affiliations:** Department of Social Dentistry, São Paulo State University (Unesp), School of Dentistry, Araraquara, SP, Brazil; University of Rochester, UNITED STATES

## Abstract

**Objectives:**

This study observed the effect of different magnification systems on dental students’ real and perceived fine motor skills.

**Methods:**

This was a laboratory-based experimental study. Students in the fifth year of an undergraduate dentistry program (N = 92) participated in this study. The dependent variables were real motor skills, perceived motor skills and time required to complete the fine motor skills test. The independent variable was the use of a magnification system under four conditions. For each condition, the Dental Manual Dexterity Assessment was performed, which consisted of inserting the #3195FF bur into targets positioned on a Styrofoam plate. The accuracy of each penetration of the targets was scored, using a point system with a maximum possible score of 246 points. Students’ perceived fine motor skills were assessed using a visual analog scale (VAS) that ranged from zero for no skills to ten for maximum skills. A descriptive statistical analysis and the repeated measures ANOVA were performed (α = 0.05).

**Results:**

The Galilean and Keplerian loupes were found to positively affect students’ real fine motor skills (p<0.01); however, perceived fine motor skills and time were significantly better (p<0.01) under the naked eye.

**Conclusions:**

Dental students’ real fine motor skills were better when Galilean and Keplerian loupes were used, but the perceived fine motor skills were not.

## Introduction

Visualization of the operating field is a major obstacle in the adoption of ergonomic posture [1]. The use of magnification systems can be a viable option for improving that visualization [2], as well as for preventing the development of musculoskeletal disorders [3]. These systems provide benefits not only for the health of professionals, but also for the quality of their work [4–9].

The magnification systems available include operating microscopes and loupes. Loupes can contain simple or multiple lenses and may be composite or prismatic. The multiple lens systems available include the Galilean and Keplerian systems. The Galilean system has concave lenses, keeps the light rays at the top of the image in the correct orientation, and produces a direct image, thus providing greater depth of field. The Keplerian system has convex lenses and produces an inverted image that must be rotated by an internal system of prisms, a process which increases the field of vision and produces greater magnification [10].

Despite the improved visualization of the operating field with the use of magnification systems, the maintenance of dentists’ fine motor skills when these devices are used has not been extensively investigated [11]. According to Bohan et al., [12] strategies used to expand the field of vision theoretically enable the execution of finer motor movements. However, the ability to perform these movements is critical since the view is magnified but the size of the field remains the same. In this situation, the physical workspace is different from its visual perception, and hand movements could therefore be incorrect [12].

Procedures in dentistry require a high level of manual skills because they require the manipulation of anatomical structures and the use of sharp instruments [13]. For this reason, an understanding of the effects of a magnified field of vision on fine motor skills is important for maintaining the quality of the procedures, particularly by students who are still in the professional training phase. In addition, the implementation of magnification when individuals already have some level of manual skills (for example, at the end of the training phase), can interfere in their perception of these skills and possibly produce resistance among operators [14].

For these reasons, the main objective of this study was to observe the effect of different magnification systems on dental students’ fine motor skills. The effect of the magnification on perceived motor skills and the time required to perform a skills test were also observed.

## Materials and methods

### Study design and sample selection

This laboratory-based experimental study was submitted to and approved by the Research Ethics Committee of the School of Dentistry of São Paulo State University (UNESP), Araraquara Campus (CAAE Registry No. 54753816.9.0000.5416). All participants signed an informed consent form.

The sample size was calculated during a pilot study and was based on the means and standard deviations of the experimental groups, with a β = 20% and α = 5%.

All undergraduate students in the fifth and final year of the dentistry program at the School of Dentistry of São Paulo State University (UNESP), Araraquara Campus were invited to participate (N = 148). Ninety-two students of both sexes, aged 18–23 years, participated in the present study (response rate = 62.16%).

These students had no experience with the use of magnification systems and were chosen because their manual dexterity had been developed over the degree program. Students in the first through fourth years were not included because their manual dexterity was still being developed.

The response variable was real fine motor skills as measured by the Dental Manual Dexterity Assessment (primary outcome), developed by Neves et al [15]. The students’ perceptions of their fine motor skills were measured using a Visual Analog Scale (VAS), and time was measured in seconds (secondary outcomes). The independent variable was the magnification system under four conditions (unaided visualization, the use of a simple loupe at 3.5x magnification, the use of a Galilean loupe at 3.5x magnification, and the use of a Keplerian loupe at 4.0x magnification). Each participant performed the manual dexterity test under the four different magnification conditions. After the completion of each test, each student answered a question on their perceptions of their fine motor skills based on a VAS.

### Magnification devices

When the tests were performed with loupes instead of under the naked eye, the loupes selected were a simple loupe by BioArt (São Carlos, Brazil), as well as a Galilean loupe, and a Keplerian loupe from the Ymarda Optical Instrument Factory (Nanjing, China).

It is important to note that the students received a short and standardized pre-training on the use of magnification loupes.

### Measurement of real fine motor skills

For the measurement of real fine motor skills, the Dental Manual Dexterity Assessment, developed by Neves et al. was applied [15].

This test was performed under artificial lighting. Each student was seated, and the testing materials rested on the work bench.

**Fig 1:**
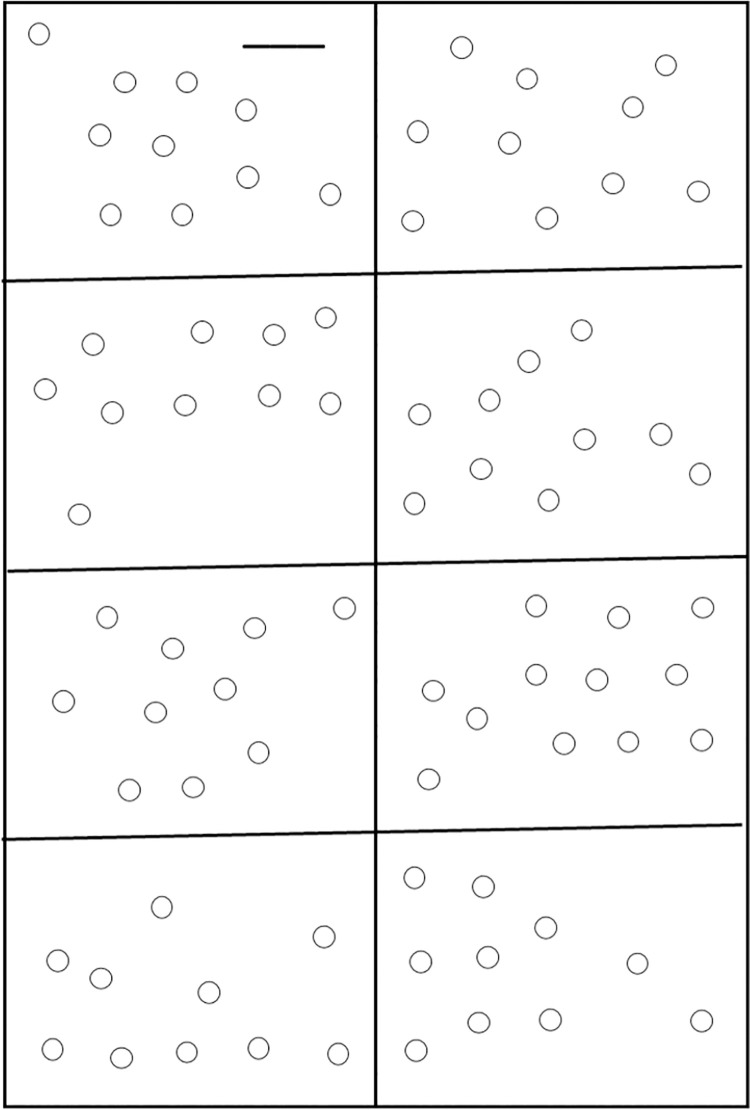
Test sheet.

The Dental Manual Dexterity Assessment consisted of the precise insertion of a bur into a series of small targets (n = 82) that had been printed on an A4-sized test sheet that was mounted under a Styrofoam plate. The test sheet consisted of a rectangle divided into eight squares containing circular targets that were 2.3 mm in diameter. The position of each target within the field was determined by a random number generator (Fig 1).

The targets were penetrated by a # 3195FF diamond bur attached to a device that simulated a high-speed, straight turbine to allow for the perpendicular entry of the bur relative to the target.

Before beginning the Dental Manual Dexterity Assessment, the students received instructions on the use and handling of the loupes, a training sheet to be used for 5 minutes to practice penetrating the targets with the different magnification systems, instructions on how to penetrate the test sheet perpendicular to its surface so that the center of each target would be accurately penetrated, the request that they attempt to penetrate all the targets on the test sheet, and instructions on the order in which they were to perform the tests: 1) using the simple loupe; 2) using the Galilean loupe; 3) using the Keplerian loupe; and 4) with unaided vision.

After the tests were performed, the classification system proposed by Neves et al.^15^ was used to score the accuracy of each penetration into the target. Scores for each penetration ranged from 0 to 3 points, with 0 being the least accurate and 3 being the most accurate. The scores were assigned according to the accuracy of the penetration, with 3 points for penetrations completely inside the target, 2 points for the penetration that touched the edge of the target and covered more than 50% of the target, 1 point for the penetration that touched at the edge of the target and covered less than 50% of the target, and 0 points for penetration that was completely outside of the target, with a maximum possible score of 246 points [17]. In cases of two penetrations touching the same target, the lowest score was counted.

The scores were given by a trained researcher, who analyzed 80 targets in duplicate in a pilot study (ĸ_weighted_ = 0.88) and who was blinded to the type of magnification used.

### Measurement of perceived fine motor skills

The students’ perceived fine motor skills were assessed using a VAS. After performing the Dental Manual Dexterity Assessment, each student was asked to draw a vertical line on the horizontal line of the VAS to represent their perceptions of their fine motor skills. The left side of the scale represented zero and no fine motor skills, while the right side represented ten and maximum fine motor skills. Each student’s line was assigned a numerical value by measuring the zero point to the point recorded by the student using a millimeter ruler.

### Time measurement

Time was measured in seconds using a timer. Measurements began the moment the student penetrated the first target and ended when the last target was penetrated.

### Statistical analysis

Data analysis were performed independently for the primary outcome (real fine motor skills) and for the secondary outcomes (perceived fine motor skills and time in seconds).

A descriptive statistical analysis was performed. The assumption of normality was met using the appropriate values for skewness and kurtosis (Sk_DMDA_ = -0.871 –-0.683, Ku_DMDA_ = -0.063–1.255; Sk_VAS_ = -1.294 –-1.209, Ku_VAS_ = -1.043–1.740; Sk_Time_ = 1.144–1.908, Ku_Time_ = 1.291–4.456). A repeated measures ANOVA was used to test the main effect of whether real and perceived motor skills differed based on type of magnification. When Mauchly’s Test of sphericity was applied neither real nor the perceived motor skills meet the assumption (p<0.01), so Greenshouse-Geisser correction was applied. For multiple comparisons, the Bonferroni post-hoc test was used. The significance level adopted in this study was 5%.

## Results

**Fig 2:**
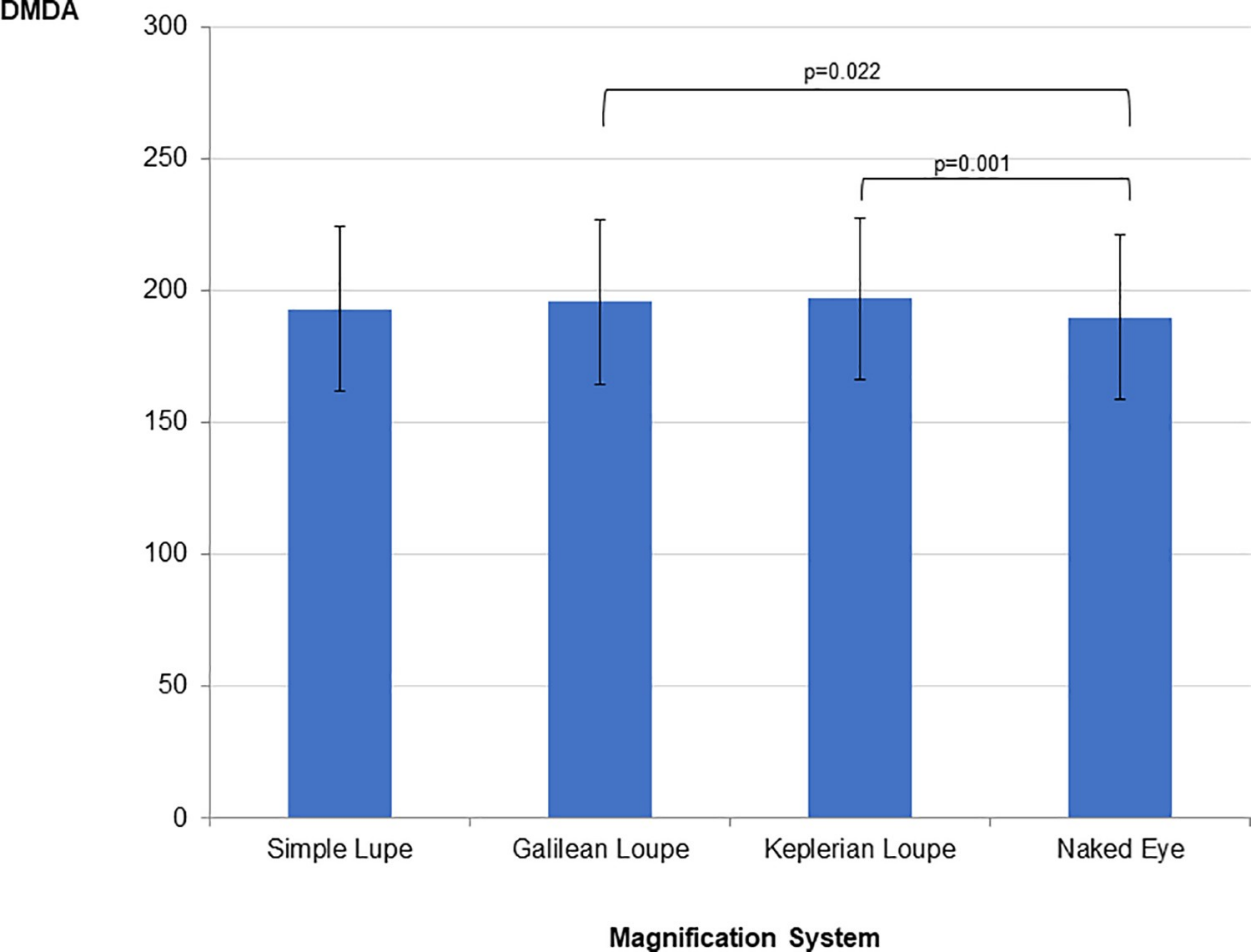
Mean, standard deviation, and summary of repeated measures Anova for the final scores of real fine motor skills as measured by the DMDA and organized by magnification system. **Repeated measures ANOVA:** SS = 2766.530; df = 2.69; MS = 1025.34; F = 6.127; p<0.01; ηp^2^ = 0.063; π = 0.945. Horizontal lines indicate statistically significant difference between means according to the Bonferroni post-hoc test.

Fig 2 presents the mean, standard deviation, and summary of repeated measures Anova for the final scores of real fine motor skills as measured by the DMDA and organized by magnification system.

**Fig 3:**
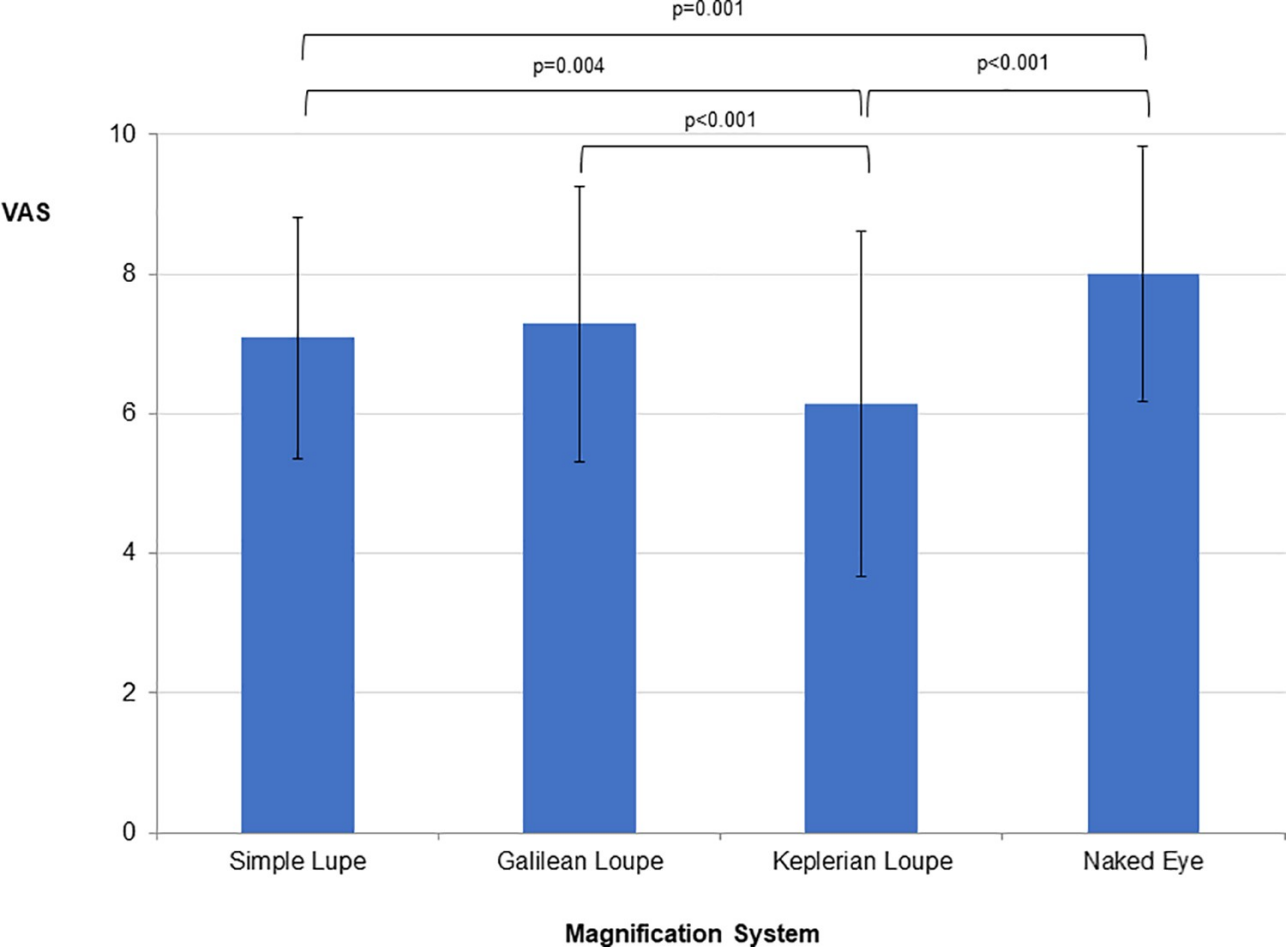
Mean, standard deviation, and summary of repeated measures Anova for the final perceived fine motor skills scores as measured on a VAS and organized by magnification system. **Repeated measures ANOVA:** SS = 160.162; df = 2.457; MS = 65.183; F = 16.345; p<0.01; ηp^2^ = 0.152; π = 1.00. Horizontal lines indicate statistically significant difference between means according to the Bonferroni post-hoc test.

The dexterity scores obtained during the use of the Galilean and Keplerian loupes were found to be similar to each other and superior to those of the naked eye. Fig 3 presents the mean, standard deviation, and summary of repeated measures Anova for the final score for perceived fine motor skills and organized by magnification system.

The scores attributed to perceived motor skills were lowest when the tests were performed under the Keplerian loupe.

**Fig 4:**
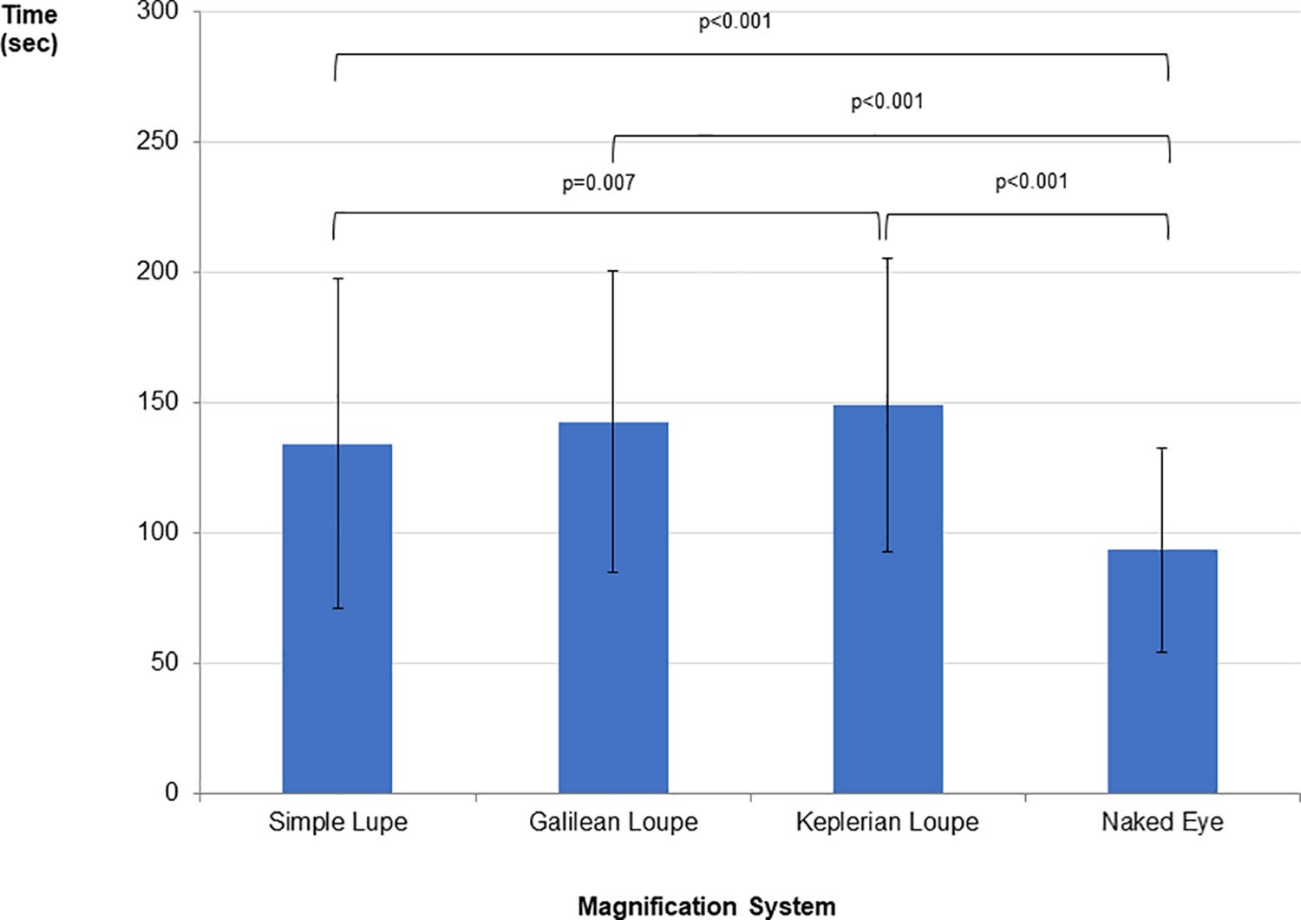
Mean, standard deviation, and summary of repeated measures Anova for the time required to complete the DMDA for each magnification system. **Repeated measures ANOVA:** SS = 172369.899; df = 3; MS = 57456.633; F = 78.466; p<0.01; ηp^2^ = 0.463; π = 1.00; Horizontal lines indicate statistically significant difference between means according to the Bonferroni post-hoc test.

Fig 4 presents the mean, standard deviation, and summary of repeated measures Anova for the time required to complete the DMDA test for each magnification system.

The students performed the DMDA in the shortest time under the naked eye.

## Discussion

The understanding of the effects of magnification devices on fine motor skills in dentistry is important for maintaining quality dental procedures. Thus, this study was performed to determine whether the use of magnification systems to amplify the operating field could negatively affect students’ fine motor skill in the professional training phase.

The results show that the magnification systems evaluated positively affected students’ real fine motor skills, regardless of the magnifying devices type used (Fig 3). These results are different of the findings reported by Congdon et al. [8] and by Mitropoulos et al., [16] who did not observe an influence of magnification on motor skills in several fields of dentistry. Other studies in the literature have also reported beneficial effects of magnification on motor skills. Bowers et al. [11] found that magnification improved fine motor skills in endodontics and could aid in the accuracy of the manipulation of endodontic instruments in the operating field. Branson et al. [17] also noticed an improvement students’ motor skills when they used loupes.

The results presented in this study support the safe use of magnification systems by individuals still in the professional training phase of dentistry programs. These devices may be beneficial, since magnification can improve many aspects of clinical and laboratory practice by helping to improve the practitioners’ visual acuity [2,4,18,19]. In addition to possible clinical benefits, magnification also provides ergonomic benefits [7,8,20,21,22,23], since the twisting and tilting of the torso and neck are not necessary if visualization is improved [24]. For these reasons, the use of magnification may reduce the risk of developing musculoskeletal disorders by reducing neck and torso strain, thus prolonging dentists’ physical health and careers [8,17,25,26]. Magnification may also compensate for presbyopia, which increases with age [23], and may decrease the ocular tension that dentists often experience when performing dental procedures [8].

In addition to real fine motor skills, perceived fine motor skills and time spent on the skills test were also evaluated. Students perceived their fine motor skills as highest when they performed the tests with naked eye; there were no statistically significant differences between the scores attributed to their perceived skills when simple loupe and Galilean loupe devices were used. It is important to note that the students who participated in this study had had no prior contact with any magnification systems. Thus, their professional abilities had been developed solely through visualization under the naked eye. It is therefore possible that the use of magnifying devices generated feelings of insecurity: though the target appeared larger, the students may have had the impression that they could not perform the movements they wanted to perform, an impression which was not supported by the scores attributed to their real motor skills (Fig 3). Their lack of experience with the equipment and the habit of using unaided vision to perform their procedures may have influenced the differences between the students’ perceptions of their skills under the naked eye and using loupes, even in the case of the simple loupe, which is easy to handle. Students perceived their fine motor skills as lowest when using the Keplerian loupe, a finding which suggests that experience more difficulty in adapting to the hand-eye coordination required of this system. This finding indicates that this magnification system is not the most suitable for the training phase.

When the time required to perform the Dental Manual Dexterity Assessment was evaluated, the tests performed with the naked eye were performed significantly faster than those performed using loupes. These results reinforce the assumption that students felt more secure with unaided vision and therefore performed the test more quickly.

In addition, the non-magnified view enabled the visualization of the test sheet as a whole, which, in turn, allowed for more immediate identification of drilled holes and places where holes were to be drilled. However, the use of loupes limited the field of vision, despite the magnification of the orifices [27]. This factor may have caused the students to spend more time locating and focusing on the target for subsequent drilling.

The data on students’ perceptions of their motor skills and on the time required to complete the test show that the implementation of loupes during the professional training phase could be important. This finding is supported by Maillet et al., [9] who emphasize that the use of magnifying lenses should begin as soon as possible in undergraduate study. Congdon et al. [8] also emphasize that learning with magnification devices early on in education programs is fundamental in helping students to avoid poor posture and to adapt more easily to magnification systems. This is because, for the development of fine motor control, an adaptation period is a necessary part of the learning process [8,9,21,22]. However, as the professional becomes familiar with magnification devices, their visual acuity improves, and the time they require to perform clinical procedures decreases [11].

A limitation of this study is the non-randomization order of the experiment. When the experiment was designed, the order in which the magnification systems would be used was defined (first, simple loupe; second, Galilean loupe; third, Keplerian loupe, and fourth, naked eye) instead of randomizing it. This decision was made because performing the exercise with the naked eye prior to the use of the magnification loupes could have interfered in the students’ perception of these systems and produce a bias in the results. Another limitation is the difference in magnification levels between simple and Galilean loupe (3.5x) and Keplerian (4.0) loupe, which may have influenced the results. However, this difference did not interfere in the results of real skills or in time required to perform the dexterity test. Despite these limitations, this study presented important results in that it quantitatively showed that the use of magnification devices positively affected the real fine motor skills of the students evaluated. However, the students’ perceived a negative effect of these systems on their skills, a perspective which may result in resistance to the adoption of this technology. Therefore, more studies must be performed in an attempt to address these limitations and to establish methods for preventing initial resistance among dental students.

## Conclusion

Dental students’ real fine motor skills were better when Galilean and Keplerian loupes were used, but the students perceived better motor skills when they worked under the naked eye than when using the magnification devices.

## Supporting information

S1 Dataset (PDF)
