# Supplementary material for: Magnification effect on fine motor skills of dental students
Source: PLoS One. 2021 Nov 8;16(11):e0259768. doi: 10.1371/journal.pone.0259768 (PMC8575251; doi:10.1371/journal.pone.0259768)
Supplement: S1 Dataset — (PDF) [file pone.0259768.s001.pdf]

Data set - Manuscript Magnification Effect on Fine Motor Skills of Dental Students

Real motor skills

ID	SIMPLE	GALILEAN	KEPLERIAN	NAKED EYE
1	149	135	205	191
2	189	210	207	205
3	216	197	227	223
4	168	145	123	126
5	99	102	98	73
6	155	119	131	129
7	185	192	188	194
8	193	180	188	182
9	121	140	141	127
10	196	192	217	189
11	167	167	166	169
12	203	182	180	188
13	206	212	213	223
14	223	236	236	220
15	238	240	228	237
16	235	239	241	241
17	229	208	222	213
18	187	185	204	205
19	192	189	188	163
20	227	185	181	167
21	242	246	244	242
22	236	229	232	217
23	220	228	228	211
24	228	215	208	180
25	183	224	214	201
26	223	232	233	238
27	221	221	221	221
28	226	221	221	204
29	213	246	234	193
30	219	199	189	185
31	219	215	228	208
32	192	216	187	186
33	214	203	186	212
34	235	233	232	234
35	206	227	221	197
36	176	172	208	170
37	226	219	222	221
38	133	116	132	147
39	143	160	147	152
40	150	163	163	155

41	157	185	177	150
42	184	178	190	197
43	178	168	154	173
44	198	228	204	208
45	185	192	188	194
46	215	209	221	192
47	198	206	223	199
48	217	222	159	180
49	196	207	218	179
50	205	212	212	184
51	201	224	227	185
52	219	216	215	218
53	220	191	205	229
54	214	211	200	201
55	207	212	196	187
56	188	182	177	177
57	231	219	229	219
58	199	200	215	201
59	207	211	213	187
60	211	219	217	188
61	206	213	209	203
62	201	195	206	212
63	157	178	180	208
64	202	218	223	207
65	211	230	220	236
66	208	222	210	211
67	235	229	229	221
68	192	213	228	205
69	218	217	204	215
70	242	240	242	239
71	168	183	185	197
72	213	217	226	227
73	218	231	236	222
74	174	193	205	196
75	204	200	201	206
76	184	188	187	200
77	206	206	197	182
78	156	184	174	180
79	159	181	196	167
80	158	158	159	114
81	165	164	165	164
82	169	164	159	171
83	163	181	196	143
84	169	172	178	147
85	205	192	182	189

86	132	151	150	131
87	143	152	160	149
88	134	124	133	148
89	124	148	153	152
90	175	173	185	187
91	156	175	180	187
92	183	192	195	174

Perceived motor skills

ID	SIMPLE	GALILEAN	KEPLERIAN	NAKED EYE
1	9.1	4.7	1.6	9.7
2	9.5	7.3	5.0	9.6
3	9.9	7.0	9.4	8.7
4	4.5	3.6	5.3	7.9
5	6.7	3.0	5.5	9.7
6	2.6	1.9	2.0	9.4
7	7.7	7.9	3.6	10.0
8	6.8	7.2	4.6	7.9
9	7.5	8.9	9.9	9.7
10	5.7	6.8	9.0	10.0
11	6.3	4.6	6.3	8.3
12	7.7	8.3	8.1	6.8
13	7.1	5.1	4.6	9.0
14	2.3	7.0	8.8	7.6
15	2.7	1.5	2.3	7.2
16	10.0	9.6	8.9	9.9
17	5.7	7.8	4.8	9.8
18	6.9	7.0	3.0	9.7
19	7.4	8.0	8.1	6.6
20	7.8	9.7	10.0	10.0
21	9.4	7.0	6.8	10.0
22	5.6	8.7	7.9	6.6
23	5.7	3.7	2.9	8.7
24	9.7	9.9	5.0	9.8
25	7.7	6.8	8.0	9.9
26	8.0	9.1	5.6	9.8
27	8.4	6.8	5.9	9.4
28	7.9	7.4	6.3	6.4
29	6.4	9.6	5.8	3.6
30	6.5	8.2	9.4	7.6
31	8.7	9.0	4.8	7.8
32	9.4	9.4	10.0	10.0

33	6.4	5.6	8.4	10.0
34	4.1	5.6	6.9	8.1
35	5.5	8.4	7.4	8.9
36	7.4	8.5	6.2	7.4
37	4.7	2.3	1.4	8.2
38	5.1	6.7	1.8	9.1
39	7.4	9.0	3.6	7.4
40	5.4	8.5	7.5	7.8
41	7.8	4.9	2.3	8.9
42	7.7	9.0	7.5	5.7
43	8.3	6.6	4.5	9.8
44	7.0	6.0	7.2	7.7
45	6.5	4.6	3.8	6.7
46	6.5	9.5	4.3	7.0
47	8.8	7.5	4.6	6.4
48	9.1	9.7	8.4	6.8
49	8.1	8.7	6.5	9.9
50	6.2	7.9	3.8	9.0
51	7.9	8.7	7.1	9.4
52	9.3	8.7	8.3	9.8
53	9.1	6.4	7.7	8.5
54	8.9	6.9	2.6	8.1
55	6.9	8.3	4.7	6.8
56	5.2	6.9	7.8	8.2
57	6.8	8.1	7.4	9.2
58	8.2	7.4	4.6	2.3
59	7.3	9.1	9.5	7.3
60	7.5	8.8	7.8	6.7
61	6.6	7.5	3.0	3.9
62	7.9	8.6	8.6	8.4
63	4.5	1.6	2.6	9.4
64	9.1	9.9	8.8	9.6
65	7.6	7.2	5.8	7.3
66	8.9	10.0	9.7	6.2
67	3.7	7.8	1.5	2.8
68	5.5	8.3	9.5	7.1
69	9.3	7.0	5.0	9.2
70	9.2	7.1	6.0	8.7
71	2.9	9.6	1.7	8.6
72	7.1	9.0	9.4	5.1
73	8.1	9.2	9.7	5.7
74	8.2	3.2	9.1	5.0
75	8.2	8.6	7.4	8.8

76	7.9	7.1	4.0	8.8
77	5.7	8.1	3.7	9.0
78	3.5	6.7	4.8	8.3
79	7.0	7.5	8.6	6.0
80	8.4	7.4	7.9	9.4
81	7.9	8.5	5.3	8.2
82	8.4	6.3	4.0	9.1
83	7.6	8.0	8.3	6.4
84	6.2	8.2	7.4	1.8
85	8.2	5.5	4.3	10.0
86	6.2	4.5	2.1	8.0
87	7.4	9.0	9.8	4.9
88	8.3	9.4	9.8	9.6
89	6.3	7.1	5.5	8.0
90	6.4	7.7	5.3	7.2
91	6.6	6.4	6.9	7.8
92	6.6	7.8	7.5	9.3

Time (minutes) spent to do the DMDA test

ID	SIMPLE	GALILEAN	KEPLERIAN	NAKED EYE
1	0.883	1.900	1.900	0.767
2	1.533	2.000	2.000	1.333
3	1.333	2.367	2.283	1.083
4	0.650	1.167	1.250	0.883
5	0.917	2.550	1.500	0.567
6	1.400	1.883	2.133	0.917
7	1.933	1.483	1.917	1.400
8	2.817	3.183	3.933	1.583
9	2.783	2.083	2.567	1.950
10	2.500	2.733	2.533	1.967
11	2.583	2.767	3.133	2.333
12	5.033	3.700	4.833	3.200
13	2.033	2.167	2.250	1.500
14	2.500	2.583	2.233	1.750
15	1.700	1.867	1.783	3.133
16	2.033	2.033	1.800	1.600
17	2.367	1.583	2.017	1.267
18	2.600	1.833	1.967	1.250
19	3.200	2.133	2.733	1.833
20	2.217	2.483	2.333	1.550
21	4.117	4.150	3.917	2.867
22	2.600	2.917	2.017	1.133

23	1.250	2.333	2.583	1.000
24	2.483	2.250	2.367	0.900
25	2.417	2.350	2.283	1.600
26	1.850	1.733	2.417	1.317
27	2.367	2.467	2.167	1.400
28	3.183	2.983	2.550	1.783
29	2.750	1.917	1.800	0.900
30	1.383	1.417	1.483	1.150
31	2.183	2.233	2.200	1.450
32	1.767	1.633	1.600	0.933
33	2.633	3.033	2.817	1.683
34	1.850	3.333	2.667	1.433
35	2.017	1.950	2.350	1.433
36	1.000	1.333	1.350	1.017
37	2.067	2.050	2.233	1.500
38	1.433	1.383	1.967	1.033
39	1.733	1.733	1.550	1.267
40	2.033	2.200	2.333	2.383
41	1.450	2.083	2.650	1.450
42	3.167	4.233	3.833	1.767
43	1.733	1.867	2.167	1.567
44	3.150	4.100	2.000	1.900
45	4.500	4.000	3.300	2.700
46	2.767	1.717	2.517	1.500
47	1.133	1.450	1.283	0.950
48	2.117	2.017	1.433	1.417
49	3.000	3.017	3.183	2.250
50	1.667	1.833	1.967	0.967
51	3.550	3.700	3.583	2.283
52	2.867	2.717	2.933	1.833
53	1.850	2.967	2.200	2.200
54	1.083	1.667	2.333	1.067
55	4.300	3.967	2.783	1.867
56	2.517	1.883	2.083	1.250
57	3.667	2.650	2.567	1.767
58	2.200	2.000	3.067	1.117
59	3.417	3.117	2.700	1.967
60	3.033	2.717	2.650	2.050
61	5.000	4.283	4.467	2.650
62	1.883	1.700	2.117	1.617
63	1.033	2.317	2.250	1.017
64	1.283	1.767	1.950	1.217
65	1.750	2.033	2.667	1.600

66	2.383	2.417	1.933	1.267
67	4.317	5.267	6.100	3.333
68	1.333	1.750	2.017	1.300
69	1.200	1.750	2.133	1.233
70	2.067	1.717	2.200	1.233
71	2.250	1.750	5.167	1.433
72	4.750	5.500	5.000	4.333
73	5.833	6.250	6.033	3.583
74	1.083	2.483	2.333	1.117
75	1.283	1.550	2.300	1.417
76	1.750	2.050	2.667	1.250
77	1.300	2.317	2.383	1.283
78	2.833	2.617	2.750	1.817
79	2.333	2.100	2.100	1.600
80	1.900	2.317	2.300	1.200
81	3.383	2.567	2.883	1.317
82	2.133	2.317	2.683	1.467
83	2.317	3.133	2.983	1.067
84	1.067	1.250	1.450	0.833
85	1.367	1.667	2.267	1.417
86	1.133	1.617	2.100	1.300
87	1.333	1.683	1.717	1.200
88	1.167	1.267	1.633	0.817
89	0.950	1.000	1.250	0.850
90	1.167	1.133	1.633	1.233
91	1.350	1.900	2.533	1.183
92	1.483	1.483	1.317	1.050
